# An Incidentally Detected Right Ventricular Pseudoaneurysm

**DOI:** 10.1155/2017/4352474

**Published:** 2017-08-24

**Authors:** Vamsi C. Gaddipati, Angel I. Martin, Mauricio O. Valenzuela, Asef Mahmud, Aarti A. Patel

**Affiliations:** ^1^Department of Cardiovascular Sciences, Morsani College of Medicine, University of South Florida, Tampa, FL, USA; ^2^Department of Internal Medicine, Morsani College of Medicine, University of South Florida, Tampa, FL, USA

## Abstract

Ventricular pseudoaneurysm is an uncommon, potentially fatal complication that has been associated with myocardial infarction, cardiac surgery, chest trauma, and infectious processes. Diagnosis can be challenging, as cases are rare and slowly progressing and typically lack identifiable features on clinical presentation. As a result, advanced imaging techniques have become the hallmark of identification. Ahead, we describe a patient who presents with acute decompensated heart failure and was incidentally discovered to have a large right ventricular pseudoaneurysm that developed following previous traumatic anterior rib fracture.

## 1. Introduction

Cardiac pseudoaneurysms are a rupture of a blood vessel or myocardial wall that is contained by pericardium, thrombus, or adhesions [1]. They differ from true aneurysms as their walls do not contain myocardial tissue and they are connected to the ventricle by a small neck (rather than forming as a broad-based outpouching). Differentiation of the two is of paramount importance for management, however, as false aneurysms have a significantly higher propensity for fatal rupture [1, 2]. This complication, conversely, has not yet been reported in the right ventricle: it is unclear if this fact is related to lower right-sided pressures (and, accordingly, wall stress) or to simple epidemiological incidence [3].

Indeed, while left ventricular pseudoaneurysms are a rare complication in and of themselves, right ventricular pseudoaneurysms are still more uncommon [4]. Following a literature review, published case reports of these right ventricular anomalies have been demonstrated to develop from lipoma [5], postcardiac surgery [6–9], following device lead extraction [10], in the infectious setting [11, 12], after remote myocardial infarction [2], as a complication of endomyocardial biopsy [3], and subsequent to penetrating chest wall trauma [13–15].

Ahead, we report one such patient who develops a large right ventricular pseudoaneurysm following a remote anterior rib fracture due to motor vehicle collision.

## 2. Case Presentation

A 64-year-old African American man presented to a university hospital with one-week history of worsening dyspnea, cough, and lower extremity edema. He endorses frequently forgetting to take his medications as well as nonadherence with dietary and fluid restrictions. Past medical history is pertinent for morbid obesity, paroxysmal atrial fibrillation (prior radiofrequency ablation, currently anticoagulated), coronary artery disease (prior stent to mid-left anterior descending (LAD) artery), hypertension, ventricular septal defect (VSD), and previous motor vehicle collision approximately ten years prior.

**Figure 1:**
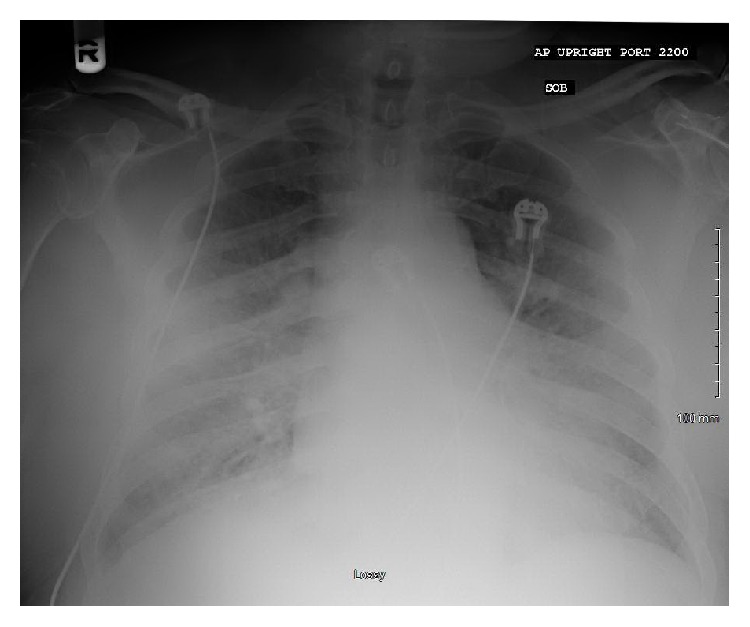
Portable chest X-ray. Minimal cardiomegaly and minimal interstitial pulmonary vascular congestion. Possible small right pleural effusion.

Upon arrival to the emergency department, he was noted to have blood pressure of 106/77 with heart rate of 94 and respirations of 23. He was saturating at 86% on 4-5 L nasal cannula and was subsequently started on Bilevel Positive Airway Pressure (BiPAP) to improve respiratory status. Physical exam was notable for bibasilar crackles, grades II/VI holosystolic murmur, mildly elevated jugular venous pressure, and 1+ bilateral lower extremity edema. Admission labs were unremarkable (including normal B-natriuretic peptide, troponin-I, and albumin). Chest X-ray demonstrated interstitial pulmonary vascular congestion (Figure 1), and EKG was sinus tachycardia with first-degree AV block. He was admitted to the cardiology inpatient service for continued management of presumed acute decompensated heart failure.

**Figure 2:**
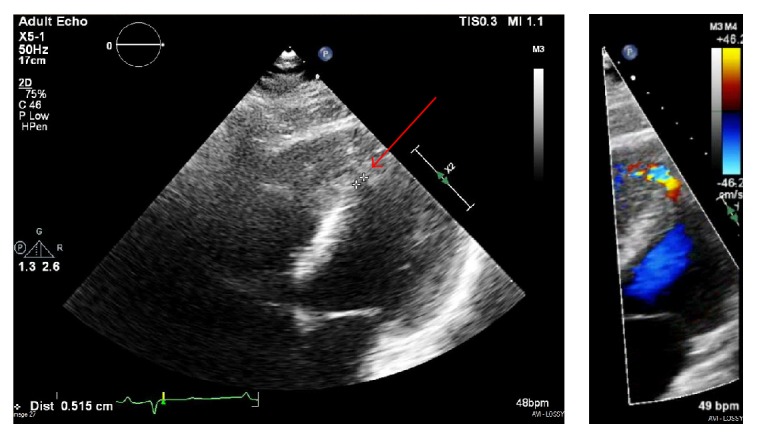
Transthoracic echocardiogram. Small, 5 mm ventricular septal defect in the distal inferoseptal/apical septum (red arrow).

**Figure 3:**
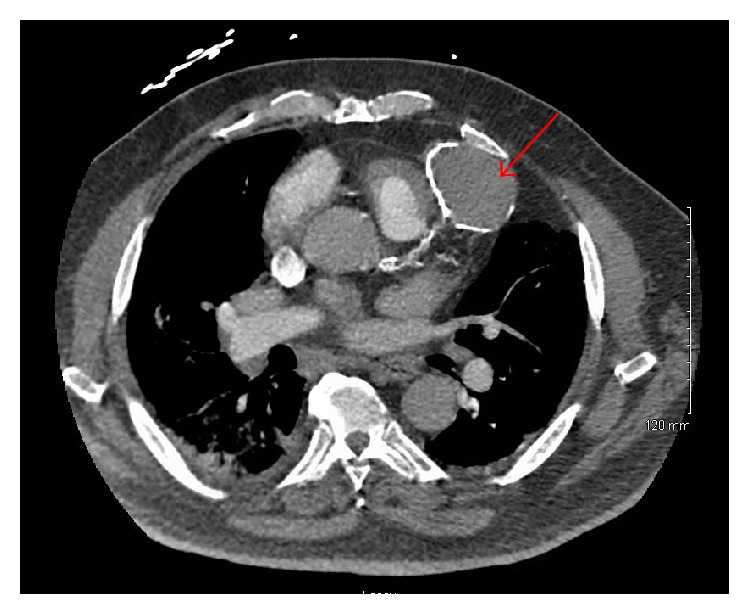
CT pulmonary angiogram.  6.4 × 4.3 cm peripherally calcified hyperattenuating lesion along the intraventricular groove highly concerning for a giant left anterior descending coronary artery aneurysm (red arrow).

Given the persistent hypoxemia, transthoracic echocardiogram (TTE) and computed tomography pulmonary angiogram (CTPA) were ordered. TTE demonstrated normal biventricular systolic function, grade 2 diastolic dysfunction, and a small, 5 mm distal inferoseptal/apical VSD (Figure 2). CTPA was without evidence of pulmonary embolism but did demonstrate a 6.4 × 4.3 cm calcified lesion concerning for giant LAD coronary artery aneurysm (Figure 3), though image quality was limited by lack of gating. Diuresis was continued with symptomatic improvement, but given these findings, the decision was made to proceed with invasive angiography.

**Figure 4:**
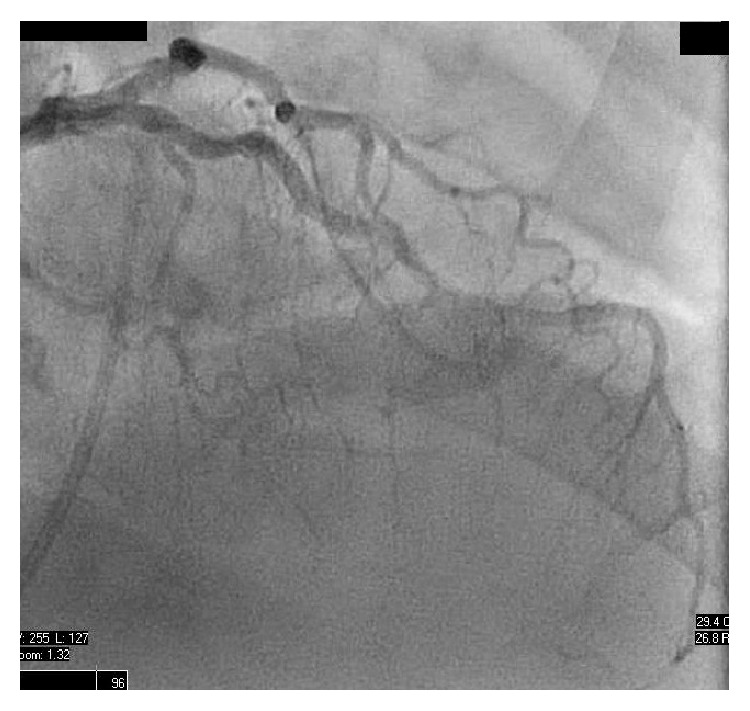
Left heart catheterization. Mild luminal irregularities of the left anterior descending artery and left circumflex arteries not requiring percutaneous intervention. No evidence of aneurysm.

Heart catheterization revealed elevated right-sided (right atrium (RA) 9 mmHg, right ventricle (RV) 52/12 mmHg, pulmonary artery 71/29 mmHg (mean 45 mmHg), pulmonary capillary wedge pressure 14 mmHg), and left-sided pressures (left ventricle 183/31 mmHg, systemic arterial pressure 171/97 mmHg (mean 128 mmHg)) without evidence of step-up intracardiac RV shunt (RA 59%, RV 61% saturation). There was elevated cardiac output (Fick—11.1 L/min (index 4.39 L/min); Thermodilution—14.4 L/min (index 5.73 L/min). Coronary angiography revealed mild luminal irregularities only, with normal ejection fraction and no evidence of LAD aneurysm (Figure 4). There was no apparent mass lesion evident with fluoroscopy and ventriculogram showed normal left ventricular wall motion.

**Figure 5:**
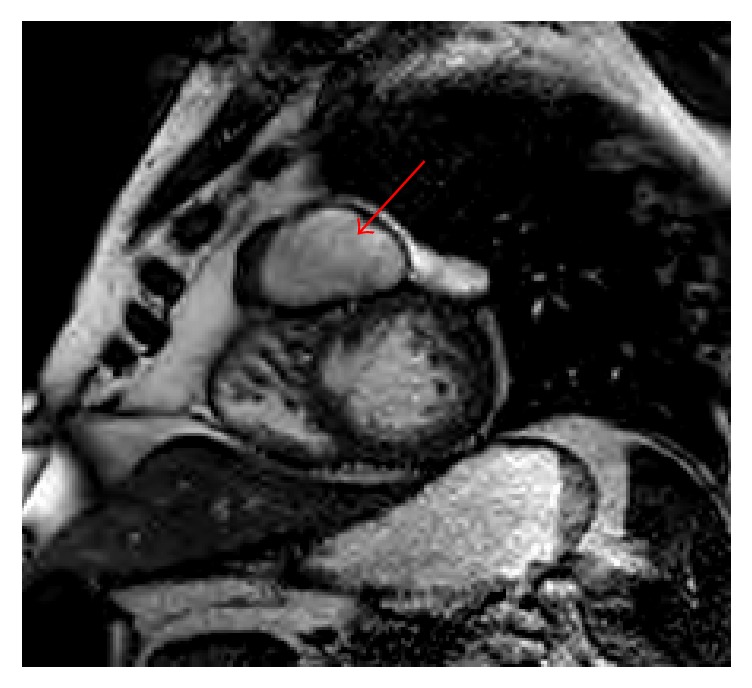
Cardiac MRI.  5.4 × 4.1 × 4.9 cm pseudoaneurysm arising from the mid-apical free wall of the right ventricle, with a 9 mm neck (red arrow). There is an adjacent muscular ventricular septal defect, which is likely related.

**Figure 6:**
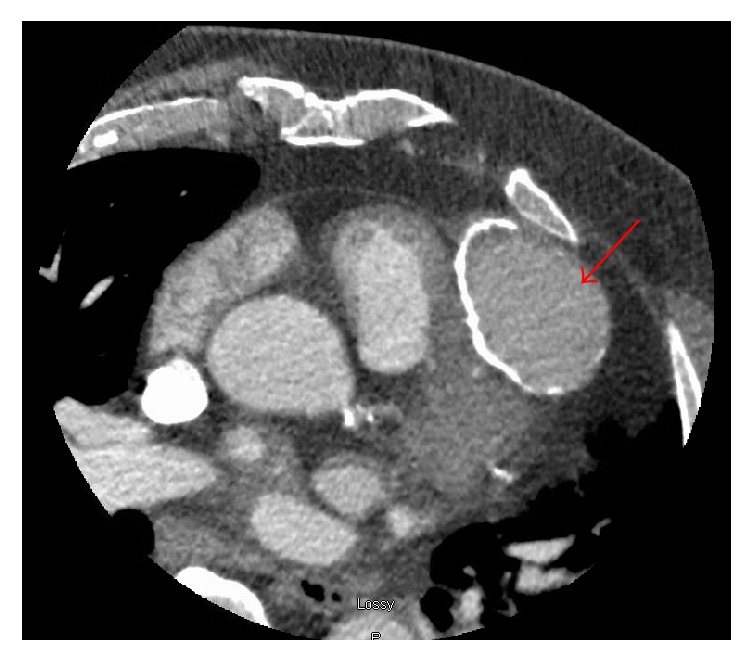
Cardiac CTA. Pseudoaneurysm arising superiorly from the mid-apical free wall of the right ventricle measuring up to 6.1 cm in maximal diameter with a 15 mm neck (red arrow).

**Figure 7:**
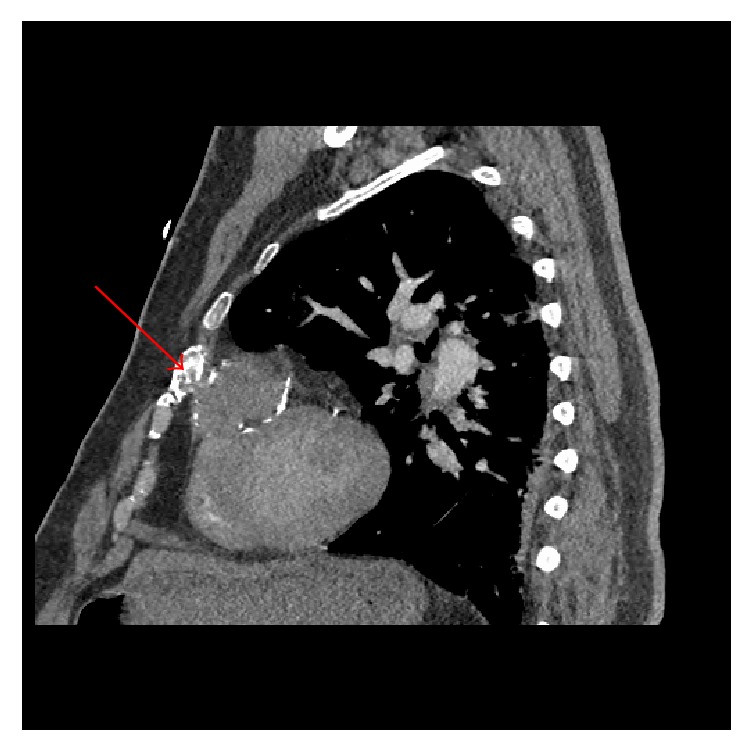
CT pulmonary angiogram. An old healed rib fracture anterior to the pseudoaneurysm (red arrow).

Follow-up imaging was performed to elucidate these findings, and cardiac magnetic resonance imaging (MRI) clarified the anomaly as a 5.4 × 4.1 × 4.9 cm pseudoaneurysm arising from the mid-apical free wall of the RV with a 9 mm neck (Figure 5). Cardiac CT angiogram was well performed and confirmed pseudoaneurysm arising superiorly from the mid-apical free wall of the RV measuring up to 6.1 cm in maximal diameter with a 15 mm neck as well as 4 × 11 mm mid-apical VSD (Figure 6). A review of the previously performed CTPA was conducted at this point and revealed a previously healed rib fracture anterior to the pseudoaneurysm (Figure 7).

Given the high output heart failure noted on angiography, an abdominal ultrasound was performed and revealed evidence of a cirrhotic liver. Upon further questioning, our patient admitted to prior longstanding alcohol abuse, now in remission. Remote motor vehicle accident was the presumed etiology to the rib fracture, and in the subsequent years, our patient developed RV pseudoaneurysm as well as VSD from the penetrating fracture. The patient was seen by cardiothoracic surgery for further evaluation, with plan to continue close surveillance per patient preference.

## 3. Discussion

Our case above describes incidental finding of RV pseudoaneurysm in a patient presenting with biventricular heart failure from elevated left ventricular filling pressures in the setting of cirrhosis and diastolic dysfunction. Initial imaging demonstrated no clear evidence of pseudoaneurysm or compressive effect upon the RV, and its discovery was contingent upon further evaluation by cardiac MRI. This imaging technique offers superior spatial resolution and tissue characterization when compared to TTE or nongated CT.

As demonstrated by this case, pseudoaneurysm lacks a typical clinical presentation, with Yeo et al. reporting that 48% of lesions are found incidentally [1]. Symptoms are varied and may include mediastinal mass effect, thromboembolism, heart failure (or other signs of low cardiac output), arrhythmia, anginal-type pain, syncope, and catastrophic rupture [1, 3, 16]. These prognosticators are nonspecific, however, and progression of disease itself is typically insidious and involves slow growth [16]. Unfortunately, given the rarity of its occurrence, there is a paucity of large scale clinical series or trials that delineate appropriate history of disease, management, and survival data. As a result, diagnosis requires adequate visualization and an individualized approach to treatment and potential intervention.

A multitude of imaging modalities exist for detection, from conventional echocardiography and contrast angiography to the novel CT and MRI techniques. Traditionally, echocardiography is a first-line test due to its wide availability and routine usage. Recognition of pseudoaneurysm, however, is contingent upon ability to visualize the ostium of the connecting orifice; continuous and pulse wave Doppler as well as color flow mapping are potential alternatives but regrettably have limited sensitivity [2, 4, 17]. Though angiography is historically the standard, cardiac CT and MRI offer new, noninvasive technique, high spatial resolution, tissue characterization, and improved visualization of segments that may be difficult to see on echocardiography [4]. In our own case, as outlined above, these procedures proved vital towards accurate diagnosis.

While optimal management remains unclear, reduction of rupture incidence is the primary goal. Towards this end, it has been reported that medical treatment of chronic pseudoaneurysm (defined as lasting >3 months) is not associated with increased risk for mortality [1]. It is possible that there may exist a stabilizing effect of previous pericardial adhesions (to contain progression of growth) in this particular cohort where acute rupture is avoided [15]. In the specific instance of right ventricular involvement following motor vehicle collision, it has as well been hypothesized that there is decreased frequency of persistent right heart failure due to decreased right-sided wall strain affording a protective factor towards morbidity and mortality [14]. As a result, the benefits of aggressive surgical intervention for asymptomatic right ventricular pseudoaneurysm remain a topic of debate. Though innovative techniques such as percutaneous closure of left and right-sided ventricular pseudoaneurysms are a possibility [18], course of action, particularly in high risk patients, should be personalized.

## 4. Conclusion

Cardiac pseudoaneurysms are a potentially fatal condition that require appropriate identification and surveillance. Given the challenge of diagnosis, index of suspicion and suitable imaging studies are a necessity for treatment. Novel cardiac imaging techniques including cardiac MRI and coronary CT angiogram should be strongly considered when clinically indicated.
